# Application of Nanoindentation and 2D and 3D Imaging to Characterise Selected Features of the Internal Microstructure of Spun Concrete

**DOI:** 10.3390/ma12071016

**Published:** 2019-03-27

**Authors:** Jarosław Michałek, Michał Pachnicz, Maciej Sobótka

**Affiliations:** Faculty of Civil Engineering, Wrocław University of Science and Technology, 50-370 Wrocław, Poland; michal.pachnicz@pwr.edu.pl (M.P.); maciej.sobotka@pwr.edu.pl (M.S.)

**Keywords:** spun concrete, micro-computed tomography, nanoindentation, deconvolution, mathematical morphology

## Abstract

The spinning of concrete is a process in which concrete mixture is moulded and compacted under the action of the centrifugal force arising during the fast rotational motion of the mould around its longitudinal axis. As a result of the spinning of the liquid concrete mixture, an element annular in cross section, characterised by an inhomogeneous layered wall structure, is produced. The heavier constituents tend towards the cross-section wall’s outer side, while the lighter components tend towards its inner side. The way in which the particular constituents are distributed in the element’s cross section is of key importance for the macro properties of the manufactured product. This paper presents procedures for investigating spun concrete and interpreting the results of such investigations, which make it possible to characterise the microstructure of the concrete. Three investigative methods were used to assess the distribution of the constituents in the cross section of the element: micro-computed tomography (µCT), 2D imaging (using an optical scanner) and nanoindentation. A procedure for interpreting and analysing the results is proposed. The procedure enables one to quantitatively characterise the following features of the microstructure of spun concrete: the mechanical parameters of the mortar, the aggregate content, the pore content, the cement paste content, the aggregate grading and the size (dimensions) of the pores. Special attention is devoted to the determination of the variation of the analysed quantities in the cross section of the element. The result of the application of the investigative procedures is presented for an exemplary spun concrete element. The proposed procedures constitute a valuable tool for evaluating the process of manufacturing spun concrete elements.

## 1. Introduction

The main aim of this study was to assess the capabilities of three different investigative methods, namely nanoindentation, micro-computed tomography (µCT) and 2D optical scanning, as applied to evaluate selected properties of the microstructure of spun concrete. In particular, it was attempted to use these methods to describe the internal microstructure of concrete, e.g. local strength parameters, pore space morphology, spatial distribution of aggregate and cement paste. The performance of utilised methods was demonstrated on the example of spun concrete samples. This type of concrete has an internal structure different from the commonly used cast in place concrete. Due to the production process, the spun concrete is characterised by a layered structure across the wall of the annular cross section. Therefore, the parameters describing the internal microstructure vary within the cross-section. Proposed methodology for determining aforementioned parameters can be a practical tool helpful, for example, in the development of the optimal technology for the production of spun concrete elements.

The spinning of concrete is a process in which a concrete mixture is shaped and compacted under the action of a normal (radial) force arising during the fast (500–700 rpm) rotational motion of the mould around its longitudinal axis. Because of its peculiarities, this method can be used to produce exclusively hollow elements. As a result of spinning, the concrete mixture introduced into the mould consolidates and retains the acquired shape as the process continues. The moulding pressure produced by spinning is not uniform across the layer being consolidated. The moulding pressure on the inner layer of the product wall is close to zero, while, on its outer side, it reaches a maximum value. A graph of the pressure across the product wall can be presented in the form of a triangle.

The generated normal pressure is initially taken by the water present between the particles of the concrete mixture. As a result, the hydrostatic pressure increases and under this pressure the water begins to displace (through the forming filtration channels directed radially towards the mould axis) from one layer to another. At the same time, the concrete mixture consolidates. The displacing water takes the finest concrete mixture constituents (mainly cement) with it. In the course of this process, the cohesion of the mixture increases and the mechanical bond between the mixture constituents acquires some strength and in certain conditions a state of equilibrium is reached, whereby the filtration stops. Since at this moment the moulding pressure is fully transferred to the solid particles of the already considerably compacted concrete mixture, the ultimate effect of the compaction will depend on the composition of the concrete mixture (to a large extent on the aggregate grading).

Under such a distribution of pressure during spinning, water is not carried away from the cross-section wall thickness equally (the element’s wall layers being characterised by different w/c values and thus by different porosity). Consequently, the mixture layers closer to the mould axis begin to consolidate only after the limit density of the outer layer is reached while the moulding pressure increases. This means that, unlike in the case of vibrated concrete, the structure of spun concrete across the element wall is inhomogeneous and layered. The structure is characterised by the fact that the heavier constituents (large particles) tend towards the cross section’s outer side, while the lighter components (cement paste) tend towards its inner side. As a result, the outer layer can become highly compact and after the concrete sets it can acquire high strength and resistance to chemical and mechanical impacts. The inner layer will consist of highly consolidated cohesive cement paste and after setting it can become highly impervious and resistant to the impact of flowing water. According to Ref. [1], the 2–4 mm thick inner layer can contain >25% more cement than the intermediate layers.

The deformation and strength characteristics of the layered structure of spun concrete have been the subject of only a few studies. Mainly macroscopic investigations of spun concrete are carried out. Marquardt was one of the first researchers [1] who described the structure of spun concrete used for the production of pipes. He experimentally found that the larger is the difference between the specific weight of the concrete mixture constituents, the faster and more completely does the mixture fractionate. For the concrete mixture, he recommended well sorted mixed aggregate particles with similar petrographic properties and a maximum diameter of 15 mm. He also described the variation in cement content across the wall of a pipe made of spun concrete. He found that cement across 83% of the wall thickness is quite uniformly distributed and an increase in cement content takes place in only the 2–5 mm thick inner layer of the cross section. To reduce cross-sectional lamination, he proposed to use layered spinning at lower mould rotational speeds. After spinning each of the layers and before feeding the next concrete mixture batch, one should pour out the spun out water from the mould and smooth the inner layer.

Achverdov, investigating the cross section of a spun concrete pipe, found [2] that the 2–3 mm thick inner layer can contain >25% more cement than the intermediate layers. The cement content in the inner layer of the cross section depends on the kind and amount of cement and on the initial water content in the concrete mixture. He also stated that the principles used for selecting a filler particle size distribution for vibrocompacted regular concretes cannot be applied to spun concretes. The total fine aggregate content should be considerable and in the case of pipes, the amount of 0/2 mm sand should constitute 40–50% by weight of the total filler content. Achverdov (similarly to Marquardt [1]) noted that, to obtain higher integrity and a higher qualitatively structure of spun concrete, it is necessary to use a multilayer moulding system. In the case of multilayer compaction, two rotation phases (the initial one and the proper one) are used for each of the layers and, when the moulding of a particular layer is finished, the centrifuge is stopped and the water drained from the mixture is removed.

Dilger, Ghali and Krishna Mohan Rao, when investigating spun concrete poles [3,4], found that, because the cross section’s inner layer has a high water and cement content, the shrinkage inside the pole’s cross section is greater than on its outside, which results in shrinkage cracks developing vertically and extending deep into the pole wall. They also found that segregation can be reduced or even eliminated through a proper concrete mixture design precluding an excess of fine-grain fractions in the concrete mixture. This means that only mortar necessary to fill the gaps between coarse aggregate particles should be supplied. Because of the high compaction energy generated during spinning, the amount of mortar is considerably smaller than in the case of normal concrete mixtures. Based on this research, it was determined that the ratio of the mass of the sand in the mixture to the mass of the coarse aggregate should not exceed 25–30%. However, because of such a low fine-grained parts content in the mixture, the latter is difficult to mix and compact in the centrifuge.

Adesiyun, Kamiński, Kubiak and Łodo [5,6] studied the influence of such parameters as sand equivalent (25–50%), cement content (410–530 kg/m^3^), plasticiser amount (0–2%), rotational speed (400–700 rpm) and spinning time (5–11 min) on the structure of spun concrete. They conducted tests on 230 mm high annular spun concrete samples with the inside diameter of 45–60 mm. The test results confirm the conclusions drawn in Ref. [3,4], concerning the difference in shrinkage between the inner and outer surface of the wall of the tested cross section. They used computer image analysis, which makes it possible to extract the information contained in the image of the cross section of the element wall, to describe the structure of spun concrete. To precisely describe the structure, the sample wall thickness was divided into twenty 2.25–3.0 mm wide strips (depending on the thickness of the cross section wall). They carried out tests for different combinations of the parameters mentioned above. Using computer image analysis, they graphically presented the distribution of aggregate, cement paste and air voids across the wall for the particular samples. They found that the aggregate content decreases from the spun concrete sample’s outer zone to its outer zone, whereas the cement paste content changes in the opposite direction (in the inner zone, the cement paste content amounts to almost 100%). The air content in the wall cross section is higher for the inner layers than for the outer ones. Composition segregation occurs in all samples. The sample with the lowest sand equivalent value (25%), the lowest speed (400 rpm) and the shortest spinning time (5 min) has the least fractionated composition. The test result confirms the ones reported in [2,3,4].

The above investigations of spun concrete mainly focus on the macroscopic description and strength and deformation tests of the sample (usually the whole sample). It is only in [5,6] that modern methods based on computer image analysis are reported, whereby the peculiar structure of spun concrete across the cross section wall can be more precisely described. However, examples reported in the literature indicate that such methods can be used to determine spatial distributions of air void [7,8,9] aggregate [7,10] or fibres [11,12,13]. Furthermore, size and shape of the mentioned constituents of cementitious composites can be characterised [14]. Thus, desiring to probe even deeper into the structure of spun concrete, the present authors used innovative (as applied to this field) investigative methods, such as nanoindentation, micro-computed tomography (µCT) and 2D optical scanning. Thanks to the use of these methods, the following features of the inner concrete microstructure were successfully quantitatively determined: the distribution of concrete components, the variation of the mortar mechanical parameters across the cross section wall and the spatial distribution of pores.

## 2. Preparation of Samples for Tests

**Figure 1:**
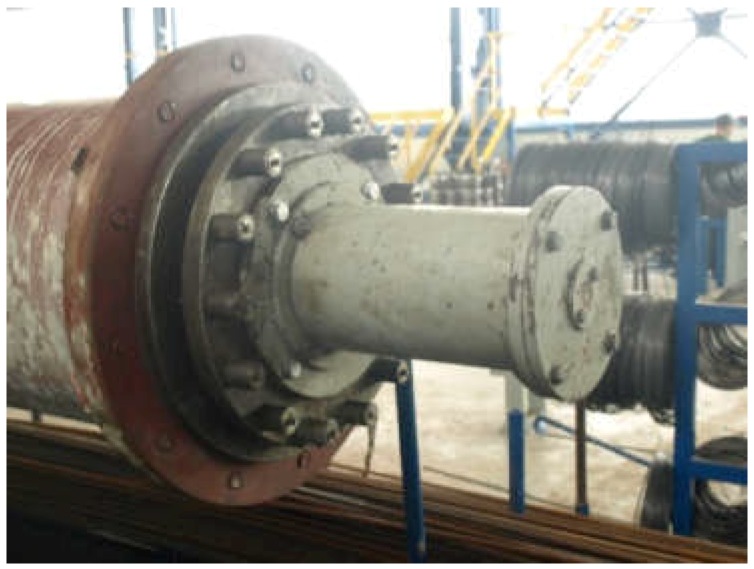
Little mould attached to steel mould used for manufacturing spun concrete power poles.

**Figure 2:**
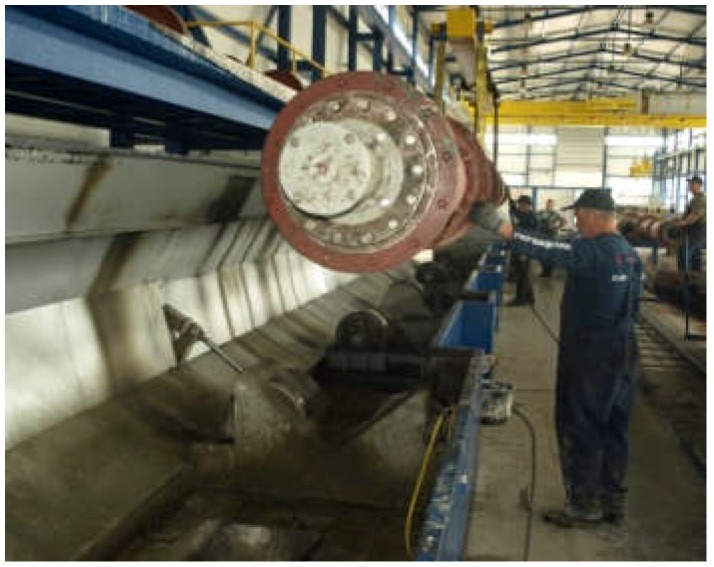
Steel mould with attached little mould is being placed in centrifuge.

**Figure 3:**
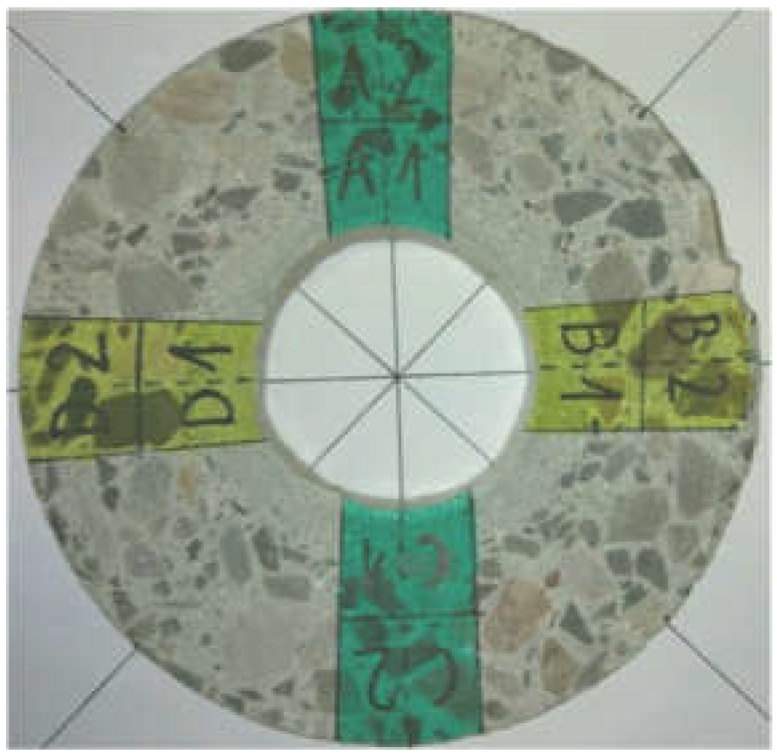
About 10 mm slice cut out of principal sample. Division of slice into smaller samples for tests proper is shown.

A sample of the concrete mixture used for the manufacture of spun concrete power poles was prepared for these investigations. The sample was made in a little mould with an inside diameter of 150 mm and a height of 300 mm (Figure 1), attached to the steel mould used for manufacturing spun concrete power poles in one of the precast concrete plant in Poland [15]. The steel mould together with the attached little mould was placed in a centrifuge (Figure 2) and subjected to spinning for 8 min at the maximum speed of 600 rpm. After concrete mixture spinning, the excess of evaporable water was removed from the little mould and the latter, together with the moulded sample, was transferred to a steam box. After about 4 h, concrete spinning steam was fed gradually (the temperature rising at a rate not higher than 10 °C/h) into the steam box. The temperature in the steam box did not exceed 60–70 °C and the concrete was cured for 8 h. Then, the supply of steam to the steam box was turned off to allow the moulds to naturally cool down to a temperature below 40 °C. Subsequently, the sample was extracted from the mould and was left in laboratory conditions for two weeks. An about 10 mm thick slice (Figure 3), in which the characteristic structure of spun concrete is visible, was cut out from the sample to be used in the tests.

**Figure 4:**
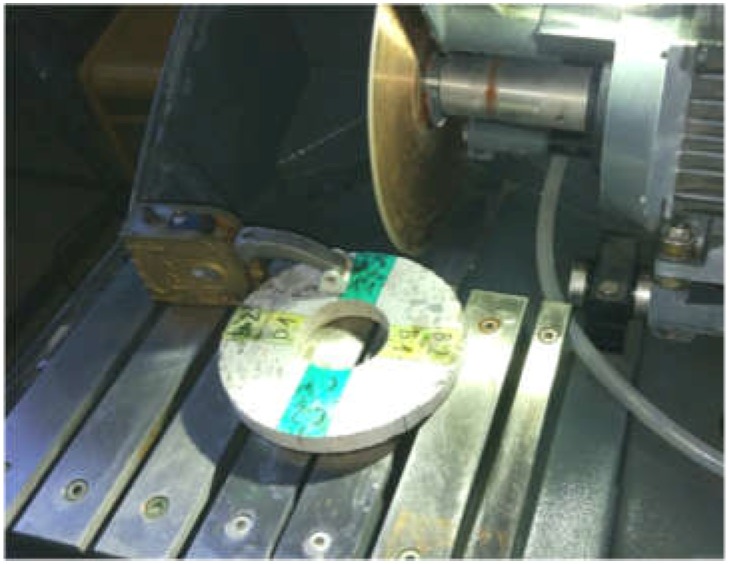
The slice mounted in the chamber of precision saw.

Prior to the tests proper, smaller samples to be tested in the particular devices were marked off on the slice (Figure 3). Samples A1–D2 (Figure 3) were tested in the nanoindenter, while the remaining part of the cross section was examined using a computer microtomograph and an optical scanner. The smaller samples were cut out using a high-speed diamond saw made by Struers Labotom-5 (Struers, Shanghai, China) (Figure 4). A series of preliminary tests was carried out on the prepared samples to determine: the indentation parameters (the force, the spacing of test points, and the necessary number of tests), the parameters of the scanning in the microtomograph (the radiation intensity, the exposure time and the filters used) and the optical scanning parameters (the way of preparing the surface and the method of imaging the tested surface). Based on the preliminary test results, a test plan was adopted. The aim of the tests was to determine the following three parameters of the tested cross section:

- the distribution of aggregate across the wall of the cross section;
- the variation of the mortar’s mechanical parameters across the wall of the cross section; and
- the distribution of pores across the wall of the cross section.

The identified quantities were related to conventional coordinate *R* in the radial direction (perpendicular to the axis of rotation of the spun concrete element), where coordinate *R* = 0 applies to the peripheral edge of the element.

## 3. Analysis of Aggregate Distribution Across Wall

### 3.1. Preparation of Samples and Scanning

The investigations presented in this section consisted in analysing the images obtained from the optical scanning of the cross section of the spun concrete element. The investigative procedure included the following steps:

1. Cutting out samples
2. Preparing their surface for scanning
3. Scanning
4. Segmenting aggregate from obtained images
5. A morphometric analysis of the aggregate based on its binary image

First, samples were cut out of the tested element and their surfaces were levelled so that they could be scanned. The most effective method of preparing the surface of the samples was sought to obtain the best result of aggregate segmentation in the next step. Different ways of grinding, etching and dyeing (using various inks and dyes (including fluorescent ones)) the matrix were tried. Ultimately, the most effective of the ways was found to be grinding, etching with 10% hydrochloric acid solution and repeated dyeing with acrylic ink and grinding again until a flat surface was obtained owing to the filling of the etched cement matrix volume with the ink. Grinding took place in a Struers Labo-Pol-5 grinding (Struers, Shanghai, China) and polishing machine using an MD Piano disc (Struers, Shanghai, China).

**Figure 5:**
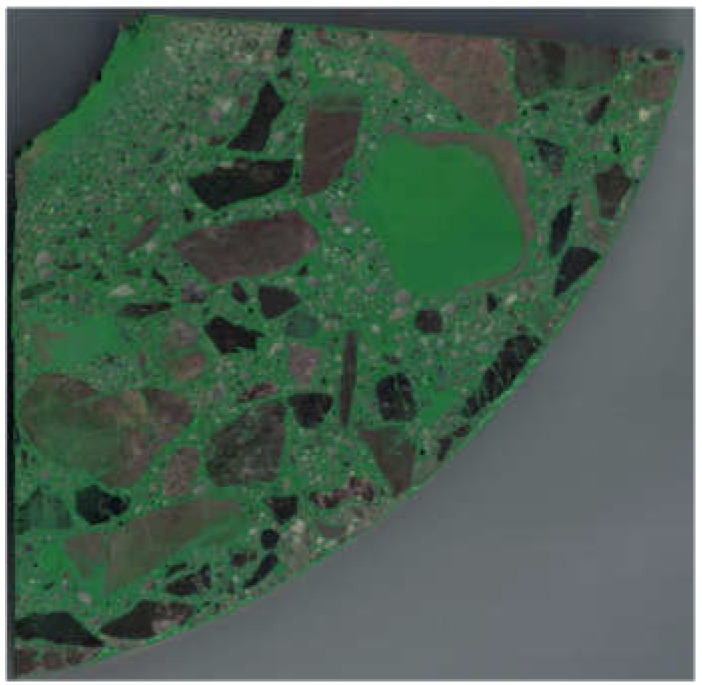
Scan of spun concrete element cross section after matrix dyeing (scale 1:1).

Scanning with a resolution of 600 dpi (which in pixel size terms amounts to 42.33 mm/pix) was carried out in a Brother MFC L5750DW device (Brother, Bridgewater, NJ, USA). The scanning result for an exemplary sample is shown in Figure 5.

### 3.2. Segmentation of Aggregate

**Figure 6:**
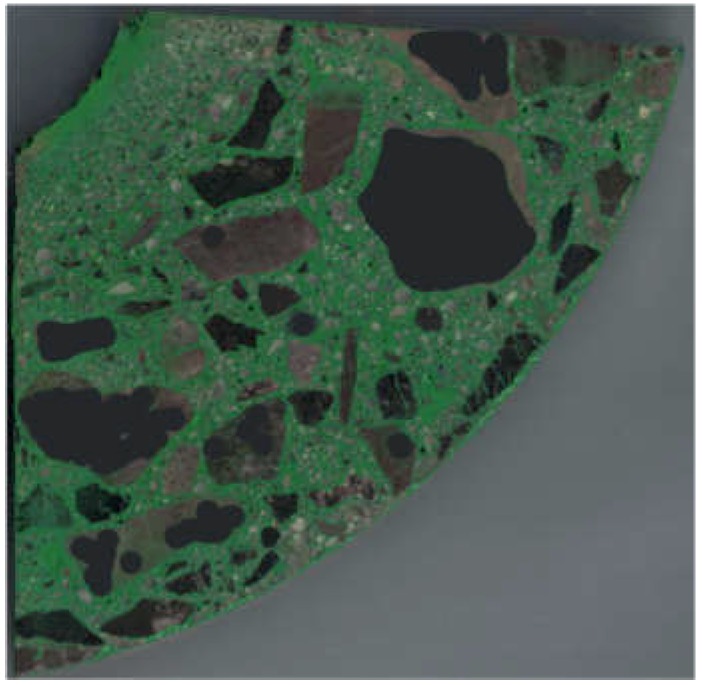
Image of sample after segmentation procedure Step 2.

The next step consisted in aggregate segmentation. Without going into details (presented below), the aim of segmentation was to obtain a binary image in which white pixels indicate the area occupied by aggregate, while pixels in the remaining area (constituting the background) are black. The aggregate segmentation procedure proposed below is based on image processing methods. GIMP 2.10.4, ImageJ 1.52e (Fiji distribution) and CTAn 1.17.1.7 + were used for this purpose. Similar to in the case of the preparation of samples for scanning, the segmentation procedure was developed through many trials. The procedure deemed the best consisted of the following steps:

1. Gaussian blurring (smoothing) with a radius of 1 pixel (3 times)
2. The manual “retouching” of large aggregate fragments that were found to be susceptible to etching (Figure 6)
3. The segmentation of the “dark” aggregate
4. The segmentation of the “light” aggregate
5. The product of the images from Step 3 and Step 4
6. The removal of “pores”

**Figure 7:**
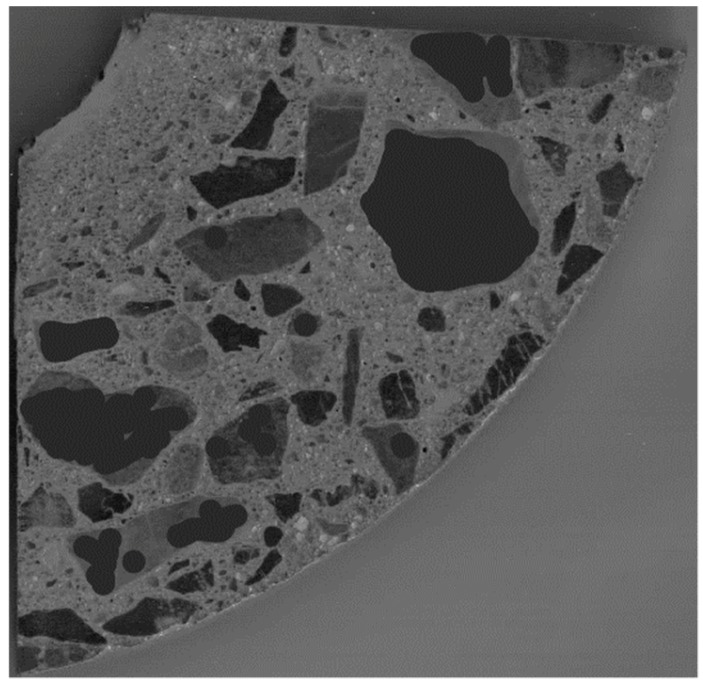
Mask created from green channel (G).

**Figure 8:**
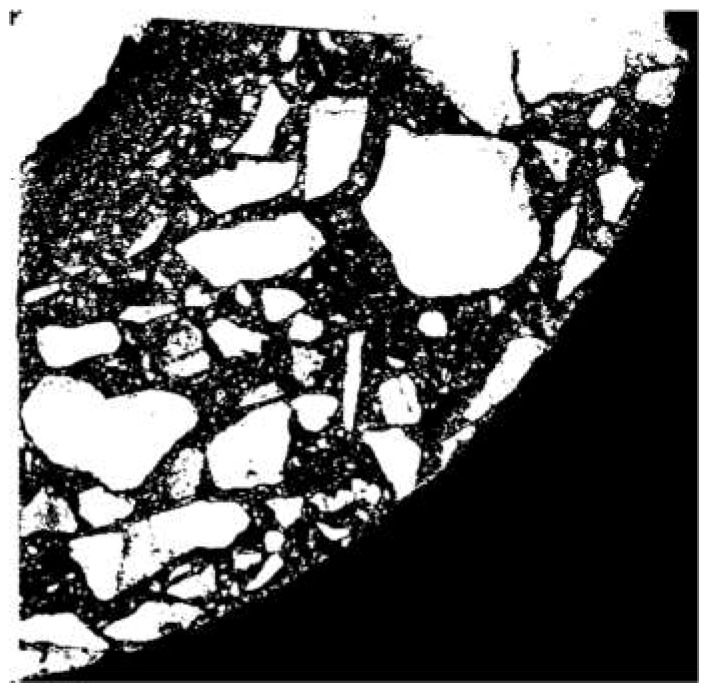
Image of sample after segmentation procedure Step 3.

To segment the “dark” (i.e., darker than the dye used in the selected RGB channel) aggregate (Step 3), first a mask was created from the green channel (G) of the image and by superimposing it on the white background a new image was created, this time in shades of grey (Figure 7). Then, thresholding was applied, assigning the value of 1 to the pixels with a brightness of 0–87/255 and the value of 0 to the other pixels (Figure 8).

**Figure 9:**
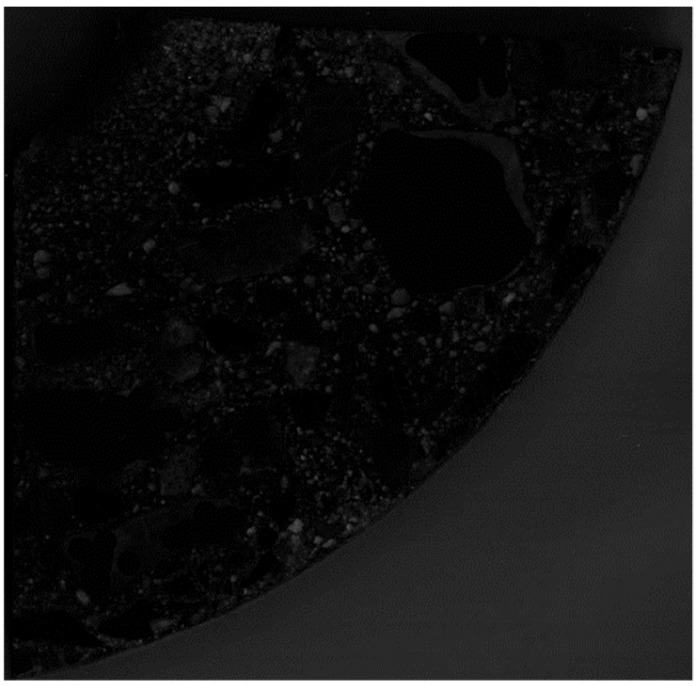
Superimposition of masks created from red (R) and blue (B) channels.

**Figure 10:**
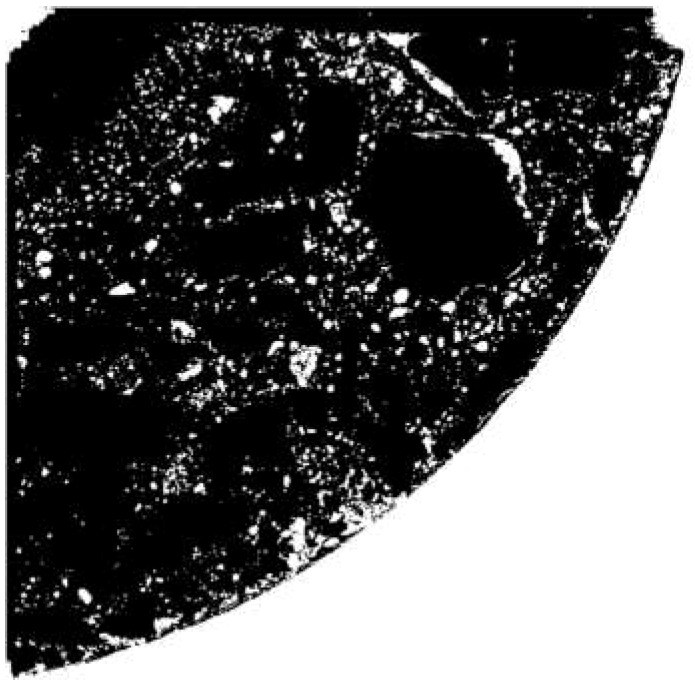
Image of sample after segmentation procedure Step 4.

The “light” aggregate (Step 4) was segmented similarly as in Step 2, this time superimposing red channel (R) and blue channel (B) masks (Figure 9). Then, thresholding according to the 20–255/255 range was applied. The result of this operation is shown in Figure 10.

**Figure 11:**
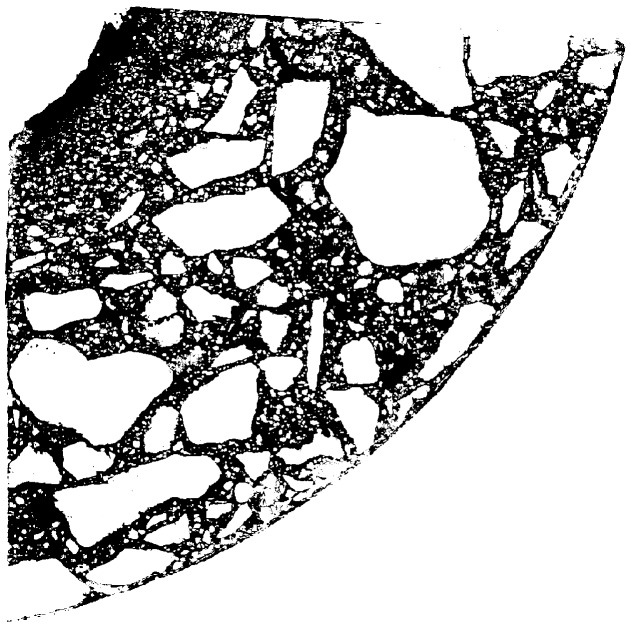
Step 5—product of images from Steps 3 and 4.

**Figure 12:**
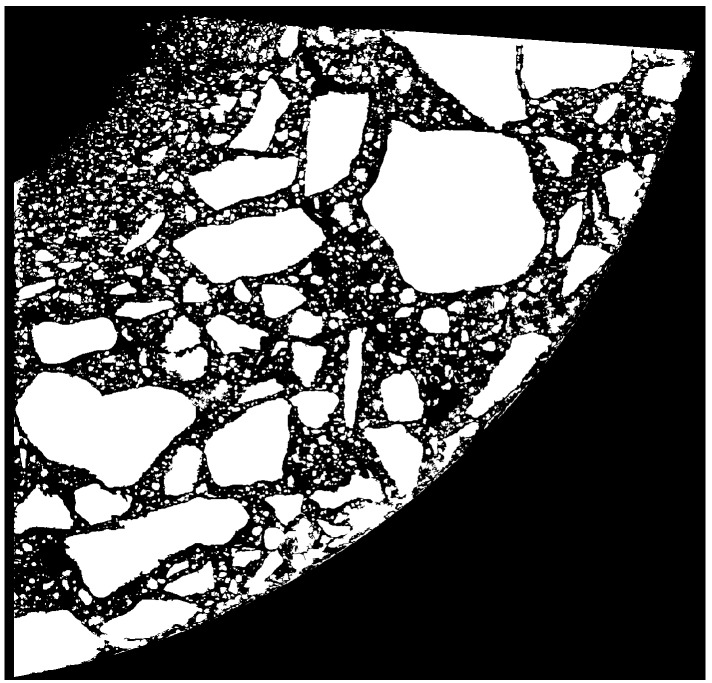
Step 6—the removal of “pores”.

The product of the images from Step 3 and Step 4 (Step 5) is shown in Figure 11. The removal of pores (i.e., black areas completely surrounded by the white area) (Step 6) and the use of a mask confining the area to the surface of the scanned sample are shown in Figure 12.

### 3.3. Morphometric Analysis

#### 3.3.1. Basic Terms and Measures

Generally, the so-called morphologic microstructure measures are used to characterise the spatial distribution of the constituents of composite materials [16]. As part of this research, the aggregate content was determined and a procedure for calculating this quantity as a function of coordinate R was proposed. Moreover, the so-called local structure thickness was used to describe the fractionation of aggregate across the wall. The methodology presented below is based on the fact that a binary image can be understood as a discrete approximation of the indicator function (as defined in [17,18,19]) on a uniform grid of rows and columns of pixels. Then, the fractional content (of aggregate in this case) in a certain considered image area Ω can be defined as the sum of pixels in the constituent, divided by the sum of pixels in area Ω.

(1) $$\phi =\frac{\sum_{\Omega \left(i,j\right)}I^{\left(k\right)}\left(i,j\right)}{\sum_{\Omega \left(i,j\right)}1}$$

where *I*^(*k*)^ is the binary indicator function of constituent *k*, *i* is the row number, *j* is the column number, and

(2) Ω(i,j)={(i,j):x(i,j)∈Ω}

stands for the number of pixels in area Ω.

**Figure 13:**
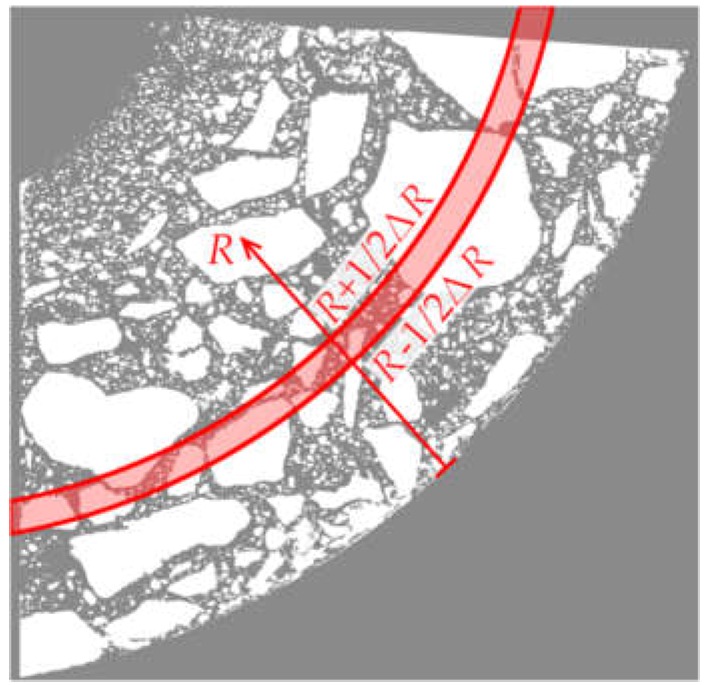
Way of selecting circumferential band.

To describe the variation in aggregate content as a function of element wall thickness *R*, a series of subareas, constituting a narrow circumferential band characterised by a fixed value of coordinate *R* in a range from *R* − 1/2∙Δ*R* to *R* + 1/2∙Δ*R*, was selected (Figure 13).

Then, the value of fractional content *ϕ*(*R*) in the band whose centre line is described by coordinate *R* can be defined as:

(3) $$\phi (R)=\frac{\sum_{\Omega_{R}\left(i,j\right)}I^{\left(k\right)}\left(i,j\right)}{\sum_{\Omega_{R}\left(i,j\right)}1}$$

where

(4) $$\Omega_{R}\left(i,j\right)=\left\{\left(i,j\right):R\left(i,j\right)\in \left[R-\frac{1}{2}\Delta R,R+\frac{1}{2}\Delta R\right)\right\}$$

runs through the set of pixels contained in the considered band. The linear dimension of a single pixel, i.e., Δ*R* = 42.3 µm, was assumed as the basic value of Δ*R* in the analysis.

**Figure 14:**
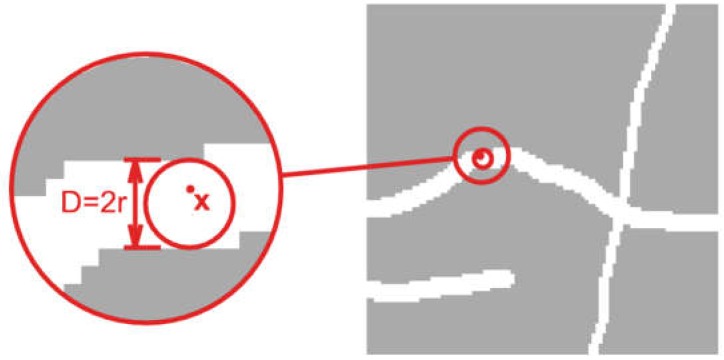
Element width in point ***x***, as maximum diameter of inscribed circle [21].

Local structure thickness is a scalar field defined in the area occupied by the considered constituent. In general, the procedure for determining local structure thickness consists in filling the area occupied by the considered constituent with circles with the possibly largest diameter. Then, the local structure thickness in given point ***x*** is defined as the largest diameter of the circle that is fully contained in the considered constituent and at the same time contains point ***x*** [20,21]. This is shown schematically in Figure 14.

Calculations are first performed for the whole image, whereby a map of the local structure thickness of considered constituent, *D*^(*k*)^(*i*,*j*), is obtained. A similar procedure as for the fractional content was used to characterise the variation in aggregate size across the concrete element wall (i.e., relative to coordinate *R*). This time the average size of the aggregate in circumferential band is defined as:

(5) $$D(R)=\frac{\sum_{\Omega_{R}\left(i,j\right)}D^{\left(k\right)}\left(i,j\right)}{\sum_{\Omega_{R}\left(i,j\right)}I^{\left(k\right)}\left(i,j\right)}$$

i.e., as the weighted average of all the *D*^(*k*)^(*i*,*j*) values of the pixels found simultaneously in band and in the aggregate. It should be noted here that the decided advantage of this way of determining aggregate size (as local structure thickness) over other methods consisting, e.g., in counting pixels, is the fact that the result in this case does not depend on an accidental “merger” of a few aggregate particles into one geometric object in the analysed image.

#### 3.3.2. Results

**Figure 15:**
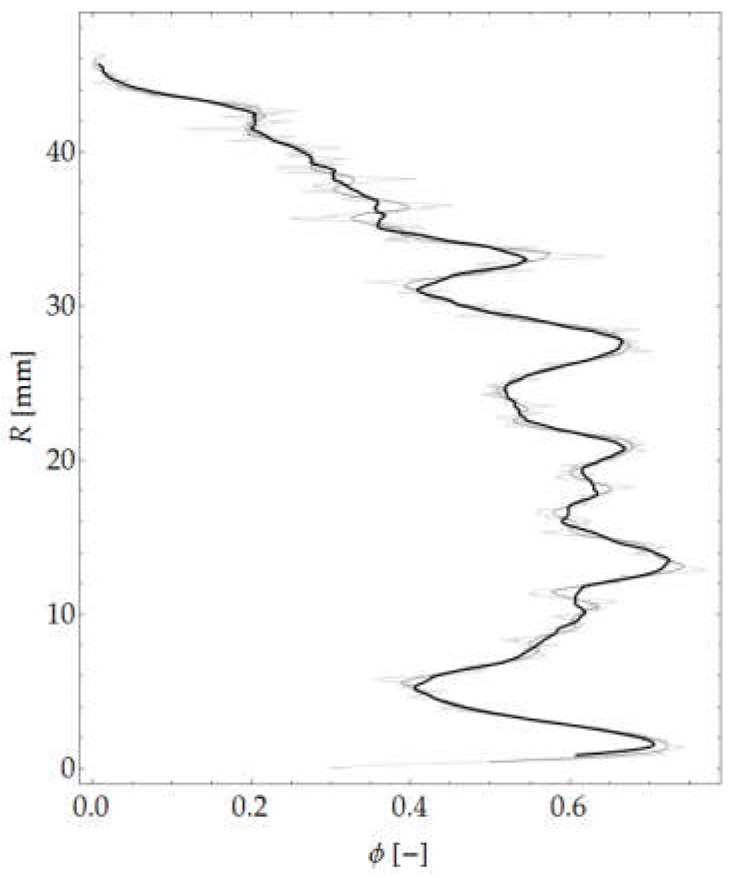
Aggregate content across element thickness.

The authors’ own procedure written in the Wolfram Language in Mathematica was used for the calculations. Selected morphological transformations were performed in the programs GIMP, ImageJ (Fiji distribution) and Bruker CTAn. A graph of the variation in aggregate content in the considered sample is shown in Figure 15.

**Figure 16:**
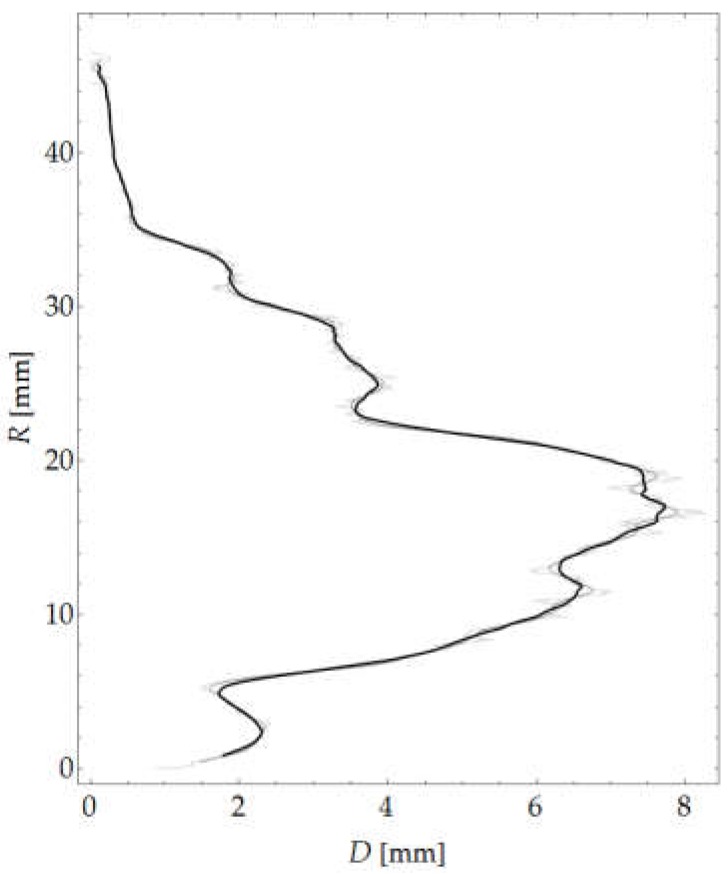
Mean aggregate size versus coordinate *R.*

**Figure 17:**
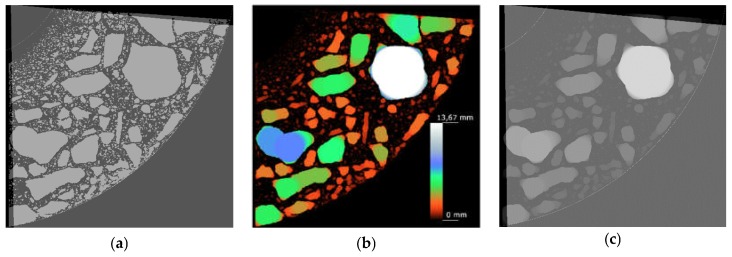
Results of applied morphological transformations: (**a**) image of sum of analysed binary image, applied mask defining ROI and outermost circumferential bands taken into account in analysis; (**b**) map of local aggregate size; and (**c**) image of sum of local aggregate size, applied map defining ROI and outermost circumferential bands taken into account in analysis.

A graph of mean aggregate size is shown in Figure 16, while a map of local structure thickness is shown in Figure 17b. Moreover, Figure 17c shows the sum of the images presenting the map of aggregate thickness, the applied ROI mask and the outermost circumferential bands taken into account in the analysis.

## 4. Analysis of Variation of Mortar Mechanical Parameters Across Wall

The nanoindentation method [22] was used to determine the mechanical parameters of the mortar, understood within the particular analysis as cement paste together with fine aggregate, i.e. finer than sand particles. Hardness (HIT), indentation modulus (MIT) and surface aggregate content (*ϕ*) in the mortar were determined on the basis of the test results.

**Figure 18:**
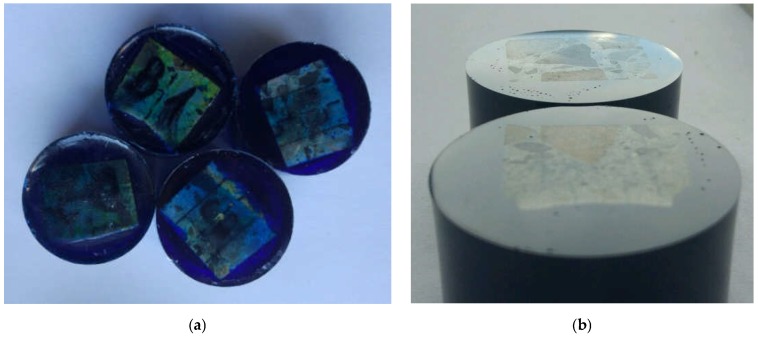
Samples for testing in nanoindenter: (**a**) samples embedded in epoxy resin; and (**b**) samples with surface polished for testing.

The concrete samples were specially prepared for tests in the nanoindenter. The preparation included: embedding concrete pieces in epoxy resin (Figure 18a) and levelling and polishing the surface tested (Figure 18b). The grinding and polishing procedure was individually fitted to each sample on the basis of the authors’ experience in the testing of concrete samples.

**Figure 19:**
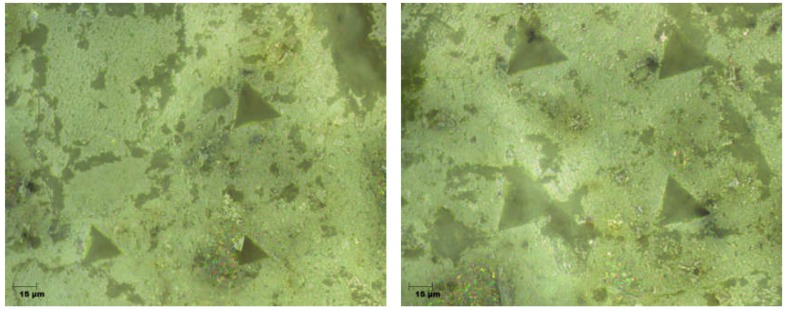
Exemplary photos of surface with trial indentations.

The proper preparation of the samples was verified by evaluating the images obtained from the optical microscope (Figure 19) and carrying out a series of trial indentations. The absence of artefacts (cracks or streaks) in the images indicated that the surface had been properly prepared for testing [23,24].

**Figure 20:**
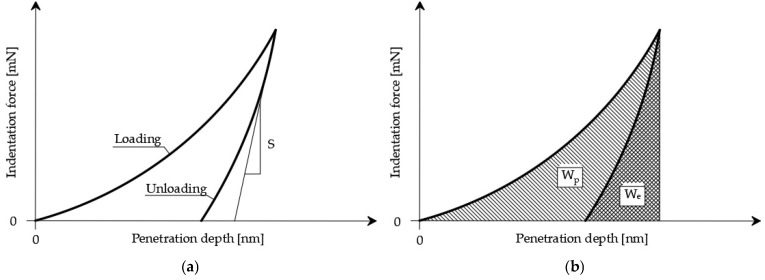
Graphical representation of standard indentation test parameters: (**a**) exemplary *F-h* dependence obtained during nanoindentation test; and (**b**) way of determining plastic behaviour (*W_p_*) and elastic behaviour (*W_e_*).

The indentation test was carried out in accordance with the standard procedure [25]. An indenter with known geometry and known mechanical parameters was pressed into the tested material and simultaneously the characteristic values of the applied load and the depth of penetration of the indenter tip were measured. In the course of the test, force *F* continuously grew, whereby penetration depth h continuously increased. Hence, the *F-h* dependence (Figure 20a) could be determined, based on which the maximum penetration depth (*h_max_*) of the indenter tip and the range of elastic *W_e_* and plastic *W_p_* deformations of the material could also be determined (Figure 20b) [22,26,27].

The basic parameters determined by the test are hardness (*H*_IT_) and indentation modulus (*M*_IT_). Hardness is defined as follows:

(6) $$H_{\mathrm{IT}}=\frac{F_{\max}}{A}$$

where *A* is a projection of the indenter contact surface onto the surface of the sample. This quantity is usually determined as a function of maximum penetration depth *h_max_* [28,29].

Indentation modulus *M*_IT_ is calculated using the Sneddon solution [30] describing the pressing of an axially symmetric rigid cone into an elastic half-space. Then, *M*_IT_ is defined as follows:

(7) $$M_{\mathrm{IT}}=\frac{1}{2}\frac{dF}{dh}\frac{\sqrt{\pi }}{\sqrt{A}}$$

**Figure 21:**
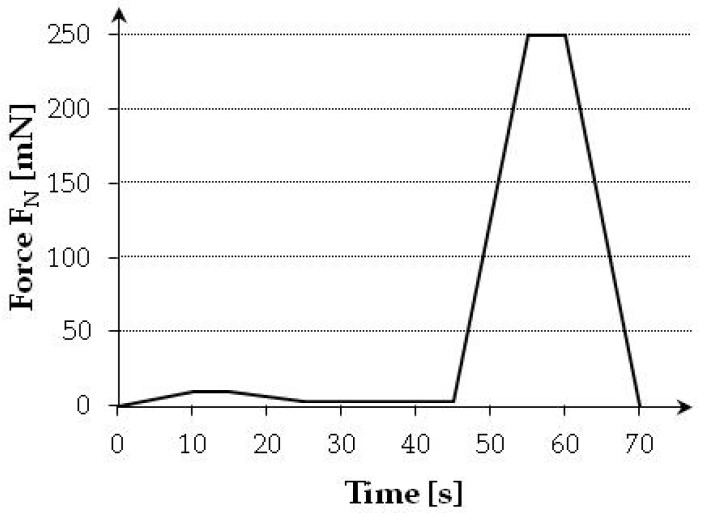
Loading function in each of the tests.

Based on trials, a loading procedure comprising two forces (10 mN and 250 mN) was adopted. The pattern of indenter loading during each test is shown in Figure 21.

The grid indentation technique (GIT) was used to determine the parameters of the sample surface [31]. According to the assumptions of GIT, penetration depth *h*, interindentation spacing *l* (mesh dimension) and number of carried out tests *N* should satisfy the following conditions (see, e.g., [32]):

3*h* < *D* < *l(N)*^1/2^ (8)

*d* < *Ω* < *h* (9)

*R_q_* < 3*h* (10)

where *D* represents the characteristic dimension of nonuniformity on a given scale, *d* is the maximum dimension of inclusions in the tested constituent, *Ω* is the characteristic dimension of the so-called representative elementary volume [33,34] and *R_q_* is the mean square deviation of surface roughness.

**Figure 22:**
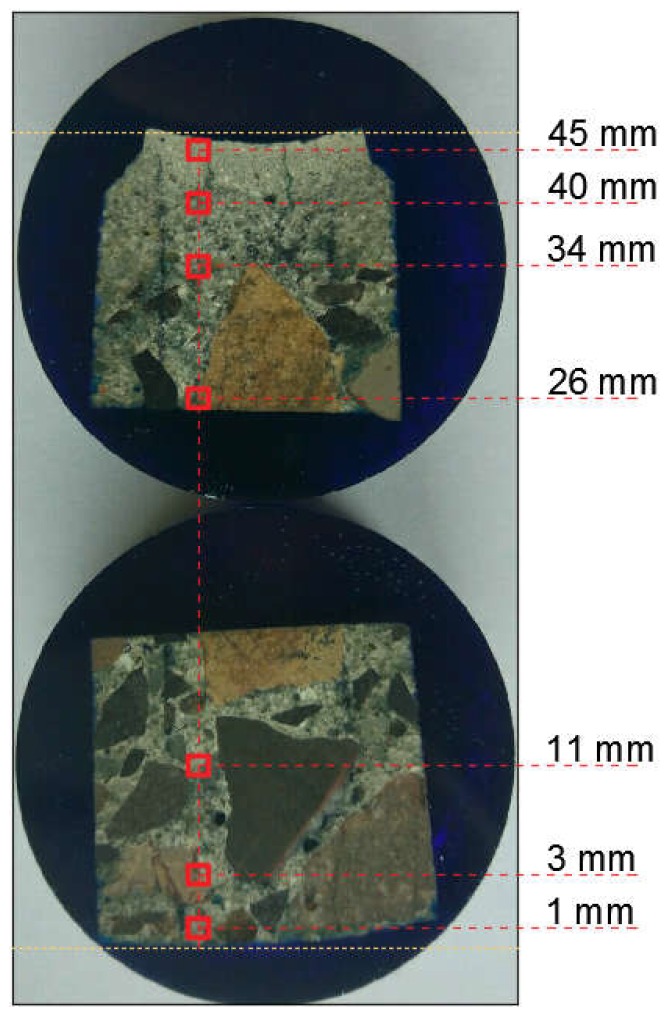
Arrangement of particular test grids—schematic representation.

Using the CSM TTX-NHT nanoindenter with the Berkovich tip [35], a series of mortar hardness measurements on seven measurement grids (Figure 22) was carried out. One hundred indentations were made at every 50 µm on each of the grids.

**Figure 23:**
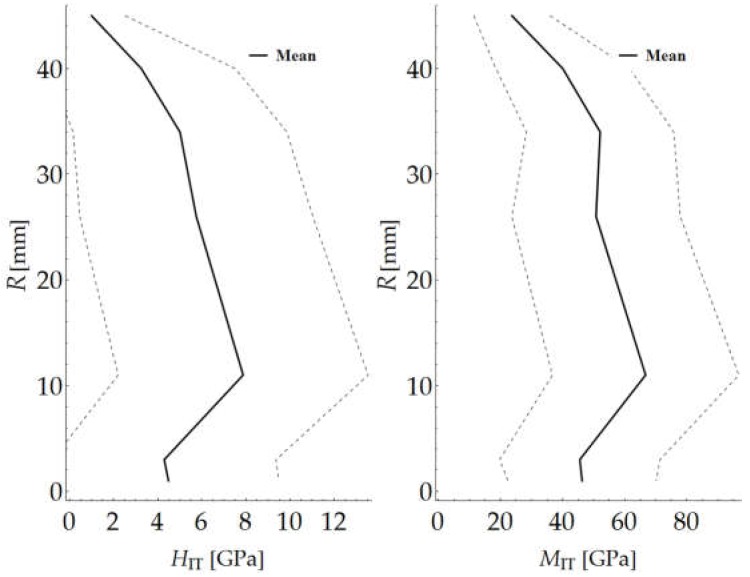
Variation in hardness HIT and indentation modulus MIT across wall of cross section.

The results of the nanoindentation tests were processed using a Mathematica script written by the authors. The mean value and the standard deviation of hardness HIT and indentation modulus MIT for the indentation force of 250 mN were determined for each of the grids. Because of the high inhomogeneity of the tested material, it was necessary to considerably increase the number of measurements to determine the parameters for the load level of 10 mN. The determined values are presented in Table 1 and in the diagrams of the variation of the mechanical parameters along radius *R* (Figure 23). The solid line marks the calculated mean value, while the broken lines correspond to the (upper and lower) boundaries of the values.

In addition, for the above measurements, the segmentation of the mortar constituents was carried out assuming two material phases (cement paste and aggregate). The deconvolution technique was used for this purpose [31,36]. According to this method, each individual indentation is considered to be an independent random event, and its results (*M*_IT_ and *H*_IT_) are considered to be random variables. The values of the cumulative distribution function for the measured *M*_IT_ and *H*_IT_ values (respectively, *F_M_* and *F_H_*) can be calculated as follows:

(11) $$\begin{array}{c} F_{M}\left(M_{\mathrm{IT}\left(i\right)}\right)=\frac{i}{N}-\frac{1}{2N}\\ F_{H}\left(H_{\mathrm{IT}\left(i\right)}\right)=\frac{i}{N}-\frac{1}{2N} \end{array}\}\;\mathrm{for}\;i\in \left[1,N\right]$$

Assuming that the distribution of the mechanical parameters of the particular constituents can be described using Gaussian distributions, expressed by mean values $\mu_{j}^{M_{\mathrm{IT}}}$ and $\mu_{j}^{H_{\mathrm{IT}}}$ and standard deviations $\sigma_{j}^{M_{\mathrm{IT}}}$ and $\sigma_{j}^{H_{\mathrm{IT}}}$, for, respectively, indentation modulus *M*_IT_ and hardness *H*_IT_, the cumulative distribution function for each segmented constituent has the form:

(12) $$F\left(X_{\mathrm{IT}(i)};\mu_{j}^{X_{\mathrm{IT}}},\sigma_{j}^{X_{\mathrm{IT}}}\right)=\frac{1}{\sigma_{j}^{X_{\mathrm{IT}}}}\frac{1}{\sqrt{2\pi }}\int_{-\infty }^{X_{\mathrm{IT}(i)}}\exp\left(\frac{-{\left(u-\mu_{j}^{X_{\mathrm{IT}}}\right)}^{2}}{2{\left(\sigma_{j}^{X_{\mathrm{IT}}}\right)}^{2}}\right)du;\;z\;X_{\mathrm{IT}}=\left(M_{\mathrm{IT}},H_{\mathrm{IT}}\right)$$

The sought values $\left\{f_{j},\mu_{j}^{M_{\mathrm{IT}}},\sigma_{j}^{M_{\mathrm{IT}}},\mu_{j}^{H_{\mathrm{IT}}},\sigma_{j}^{H_{\mathrm{IT}}}\right\}$, *j* = 1, *n* are determined by minimising the difference between the cumulative distribution function for the experimental results and the one assumed in the form of a Gaussian distribution, i.e.,

(13) $$\begin{array}{l} \min[{\sum_{i=1}^{N}\left(\sum_{j=1}^{n}f_{j}F\left(M_{\mathrm{IT}(i)};\mu_{j}^{M_{\mathrm{IT}}},\sigma_{j}^{M_{\mathrm{IT}}}\right)-F_{M}\left(M_{\mathrm{IT}\left(i\right)}\right)\right)}^{2}\\ +{\sum_{i=1}^{N}\left(\sum_{j=1}^{n}f_{j}F\left(H_{\mathrm{IT}(i)};\mu_{j}^{H_{\mathrm{IT}}},\sigma_{j}^{H_{\mathrm{IT}}}\right)-F_{H}\left(H_{\mathrm{IT}\left(i\right)}\right)\right)}^{2}] \end{array}$$

**Figure 24:**
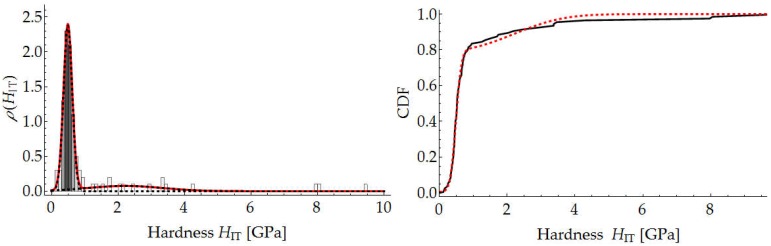
Segmentation of mortar components through deconvolution based on hardness histogram.

**Figure 25:**
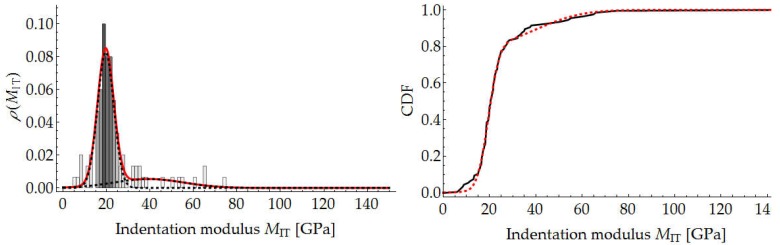
Segmentation of mortar constituents through deconvolution based on indentation modulus histogram.

Thanks to this approach, one can estimate the aggregate content in the mortar. An exemplary result of segmentation for one measurement grid is shown in Figure 24 and Figure 25.

**Figure 26:**
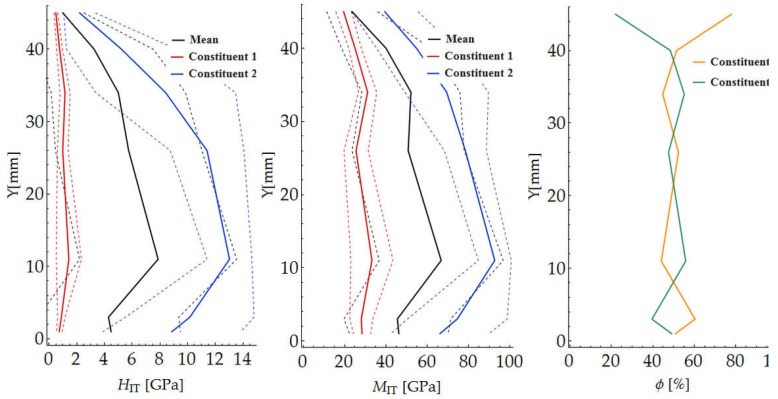
Variation in hardness H_IT_, indentation modulus E_IT_ and constituent content across wall.

The segmentation results for the whole sample are presented in Table 2 and in the diagrams of the variation of the mechanical parameters along radius *R* (Figure 26).

## 5. Analysis of Distribution of Pores Across Wall

**Figure 27:**
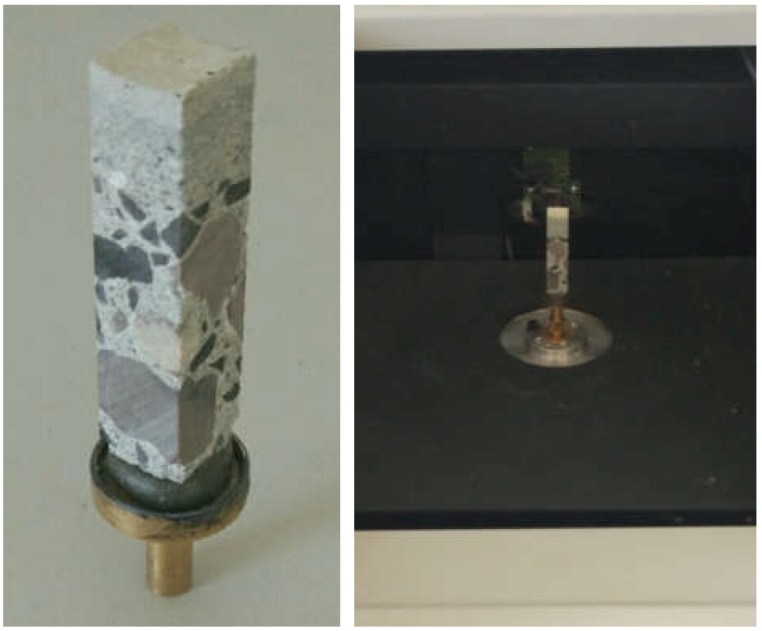
Sample mounted on base and placed in microtomograph chamber.

The non-destructive technique of micro-computed tomography (µCT) was used to characterise pore space variation. The adopted approach was similar to those presented in [37,38,39,40]. The sample was trimmed to a rectangular prism whose dimensions ensured the analytical resolution of 10 µm/pix. Then, the rectangular prism was mounted on a base and placed in the microtomograph chamber (Figure 27).

**Figure 28:**
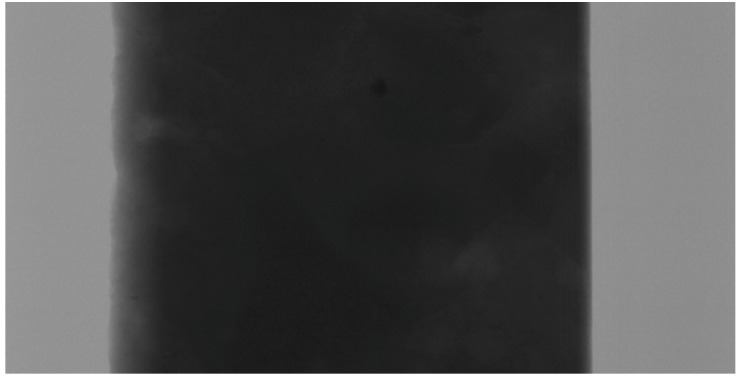
Exemplary projection of material structure.

Scanning was carried out using the Bruker Skyscan 1172 scanner. The scanning consists in subjecting the prepared sample to a series of exposures and reconstructing the material structure from the obtained projections (Figure 28).

The scanning parameter values shown in Table 3 were selected on the basis of trial scans and the earlier tests of the samples.

The NRecon program based on the Feldkamp algorithm [41,42] was used for image reconstruction. The set of reconstruction parameters is shown in Table 4.

**Figure 29:**
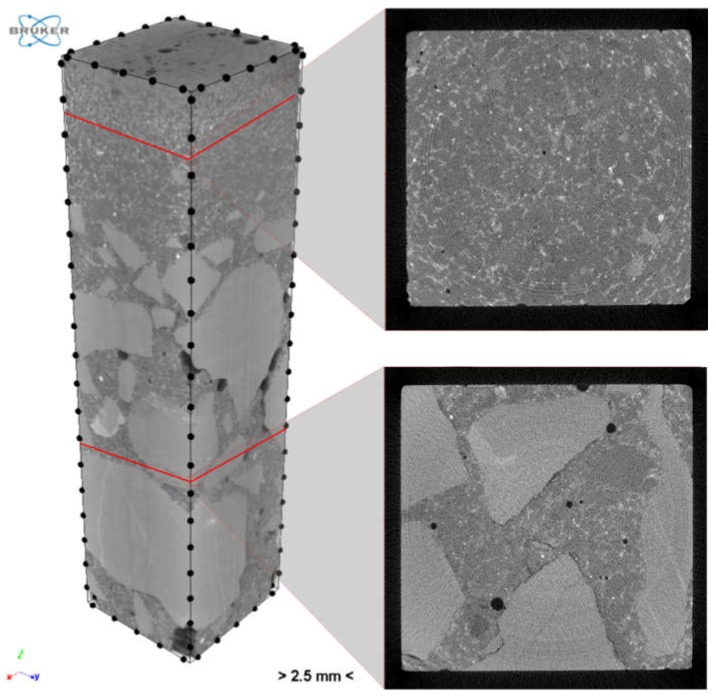
Reconstructed structure of sample with exemplary cross sections.

**Figure 30:**
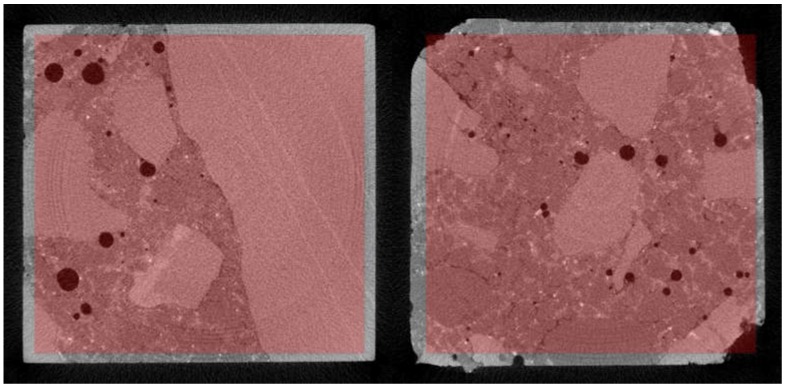
Region of interest (ROI) for selected cross sections.

The reconstructed structure of the tested sample is shown in Figure 29. To quantitatively and qualitatively evaluate the material structure, one should additionally determine the “volume” of interest (VOI) in the reconstructed model. A rectangular region of interest, marked red in the exemplary cross sections of the sample (Figure 30), was adopted.

The obtained results were analysed using the Bruker software (CTAn and CTVox) and a Mathematica script written by the authors.

**Figure 31:**
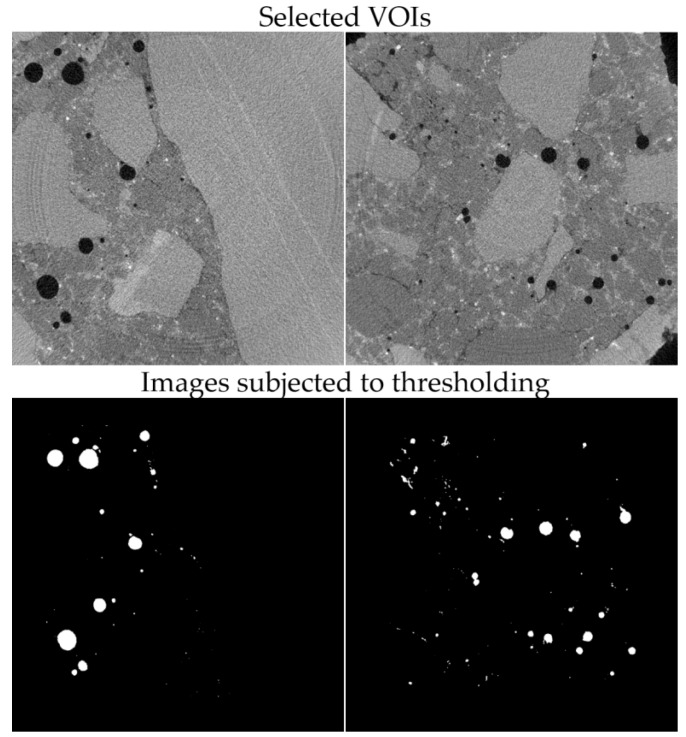
Segmentation of pores for selected cross sections.

To estimate the porosity of the tested material, it was necessary to segregate the pore space from the images. For this purpose, the images were binarised (using thresholding) for the particular cross sections. An exemplary result of the segmentation for selected cross sections is shown in Figure 31.

**Figure 32:**
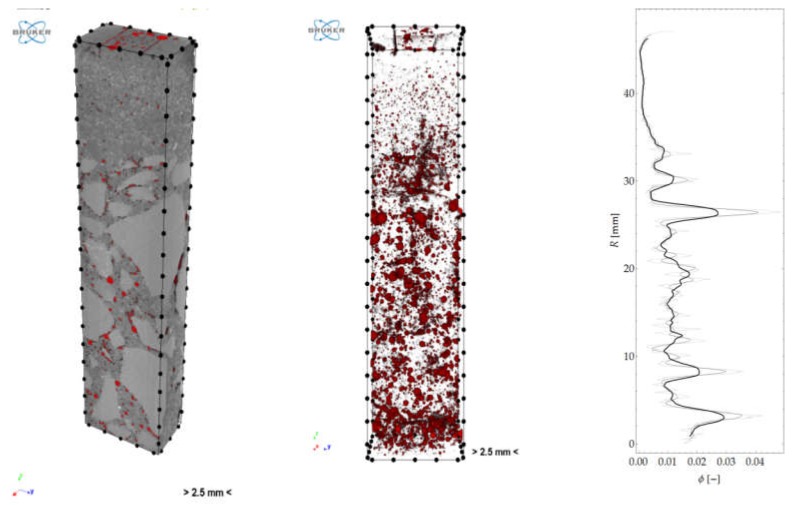
Cross section through 3D model of concrete sample with detailed pores, and pore space within VOI.

**Figure 33:**
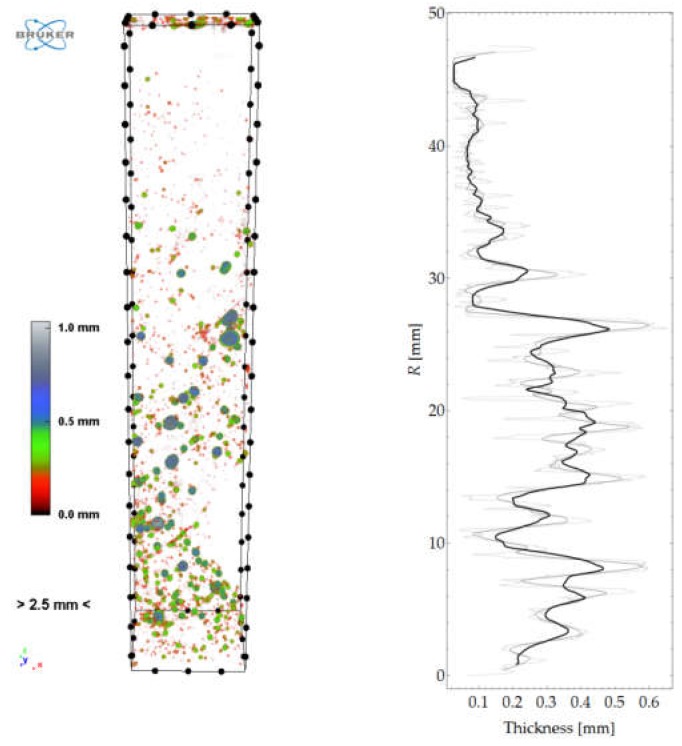
Spatial distribution of pore “thickness” and graph of mean thickness along sample height.

The isolated pore space could be presented in a 3D model and quantitatively analysed (Figure 32). The graph in Figure 32 represents porosity versus sample height. Figure 33 shows the spatial distribution and a graph of pore local thickness versus sample height. It should be noted that pore thickness is understood here similarly as described in Section 3.3. However, this time, the calculations were performed as three-dimensional and local thickness was defined as the maximum diameter of a sphere contained in the pore volume.

**Figure 34:**
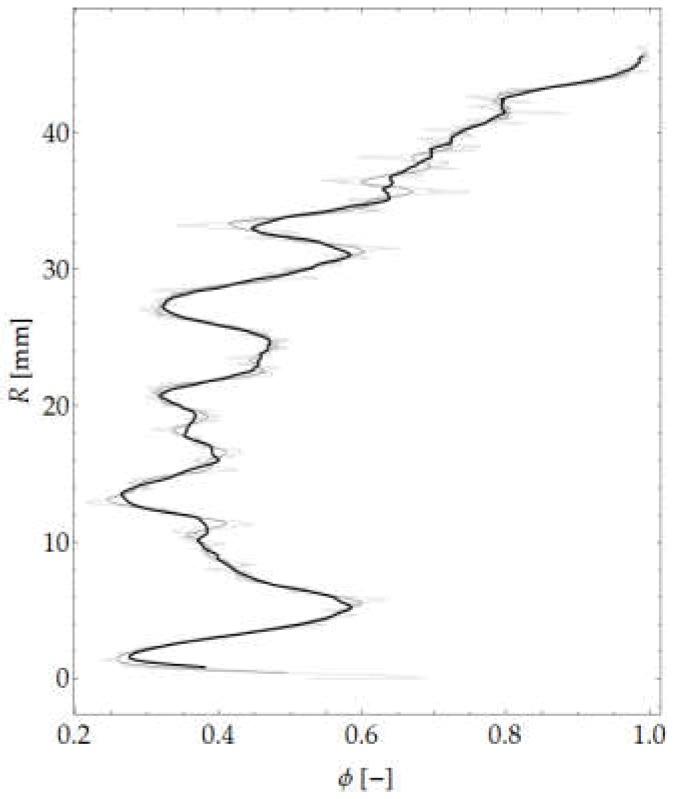
Graph of mortar content along sample height.

Considering the results obtained from 2D and 3D scanning and knowing the aggregate and pore content, the cement paste content was calculated. A graph of this quantity is shown in Figure 34. In this case, it was assumed that the analysed material consisted of three constituents, i.e., aggregate, mortar and pores.

## 6. Discussion

A test methodology comprising three different testing methods, namely nanoindentation, micro-computed tomography (μCT) and two-dimensional optical scanning, is presented. The methodology was used to describe selected features of the inner microstructure of spun concrete, such as the distribution of its constituents, the variation of the mechanical parameters of the mortar across the cross-section wall, and the spatial distribution of pores. Tests were carried out on a sample moulded from the mixture used for the manufacture of spun concrete power poles in one of the precast concrete plants in Poland.

The variation of the aggregate content and the mean aggregate size across the wall of the cross section of a spun concrete sample was characterised using an aggregate segmentation procedure based on image processing methods, and a morphometric analysis employing mathematical morphology. The aggregate segmentation method is similar to the computer image analysis method used in [5,6]. The obtained results are satisfactory, providing not only a qualitative, but also quantitative description of the distribution of aggregate across the wall of a spun concrete cross section.

Thanks to the use of the nanoindentation method, it was possible to determine the mechanical parameters of the mortar (cement paste and fine aggregate) across the wall of the cross section of a spun concrete sample. The hardness (HIT), the indentation modulus (MIT) and the surface concrete content (ϕ) in the tested mortar were determined. Because of the size of the nanoindenter tip and the indentation forces used, testing was limited to places where there was no coarse aggregate in the sample concrete. This means that acquired information about the mechanical characteristics of spun concrete across the wall of the cross section is not full. However, in addition to the measurements of the mechanical parameters, the authors succeeded in segmenting the mortar constituents (cement paste and aggregate) using the deconvolution technique. Based on the obtained results, it was possible to trace the distribution of cement paste (and thus of cement) across the wall of the cross section. The findings reported by Marquardt [1] that cement is quite uniformly distributed across the wall and that only in the inner layer of the cross section its content increases were confirmed.

The variation in pore space was characterised using micro-computed tomography (µCT). Thanks to this method, it was possible to spatially (in 3D) describe the distribution of pores in the sample as well as their distribution across the wall of the cross section. Mainly closed air voids were found to occur. The results of the analyses are surprising and they do not corroborate the literature results (e.g., [5,6]), where it is suggested that the air content in the cross section of the wall is rather higher in the inner layers than in the outer layers.

The investigative methodology comprising nanoindentation, micro-computer tomography (µCT) and two-dimensional optical scanning seems to be useful for evaluating selected features of the internal microstructure of spun concrete. The methodology allows one to gain an insight into the interior of concrete and it yields qualitatively and quantitatively valuable results. The developed procedures for investigating the internal structure of spun concrete can be successfully used, e.g., to evaluate the process of manufacturing spun concrete elements, by verifying the porosity of the produced material, the fractionation of the aggregate, and the cement content, taking into account the variation of the parameters across the wall.

Taken together, the main contribution of the work to the field is the development of a procedure for the quantitative description of selected microstructure parameters. It should be emphasised that special attention was paid to the evaluation of the variability of these parameters in the cross section of the element. In the future perspective, the developed procedure will serve as a base for upcoming studies planned by the authors. For instance, the procedure allows for a comparative assessment of elements manufactured under different technological regimes. Thus, the methodology proposed in the article may prove to be a useful tool in the optimisation of manufacturing process concerning, i.e. selection of time and speed of mould spinning. Moreover, the procedure can be utilised in the analysis of the spun concrete microstructure changes along the traction pole, for which the diameter decreases towards its top.

## Figures and Tables

**Table 1:** Mean values and standard deviations of measured hardness H_IT_ and indentation modulus M_IT_.

R	µM_IT_	σM_IT_	µH_IT_	σH_IT_
(mm)	(GPa)	(GPa)	(GPa)	(GPa)
1	46.26	23.86	4.49	5.00
3	45.52	25.83	4.29	5.07
11	66.72	30.00	7.87	5.67
26	50.76	27.12	5.75	5.28
34	52.09	23.75	5.01	4.84
40	40.04	21.53	3.26	4.26
45	23.66	12.31	1.00	1.53

**Table 2:** Hardness H_IT_, indentation modulus E_IT_ and fractional content *ϕ* of tested material constituents.

R	Constituent	µM_IT_	σM_IT_	µH_IT_	σH_IT_	ϕ
(mm)		(GPa)	(GPa)	(GPa)	(GPa)	(%)
1	1	28.42	4.16	0.74	0.21	50.90
	2	66.13	23.69	8.87	4.98	49.10
3	1	28.05	5.53	0.89	0.26	60.40
	2	74.51	24.12	10.16	4.64	39.60
11	1	33.23	10.17	1.43	0.91	44.10
	2	92.63	7.99	13.02	1.62	55.90
26	1	25.54	5.88	0.99	0.39	52.40
	2	78.44	10.14	11.41	2.66	47.60
34	1	31.12	4.38	1.16	0.36	44.80
	2	69.36	20.21	8.42	5.04	55.20
40	1	25.06	3.04	0.77	0.22	51.50
	2	54.80	19.31	5.23	3.97	48.50
45	1	19.52	3.76	0.49	0.13	78.00
	2	39.42	16.14	2.21	1.15	22.00

**Table 3:** Selected major scanning parameters.

Parameter	Value
Source Voltage (kV)	100
Source Current (µA)	100
Image Pixel Size (µm)	9.88
Filter	Al + Cu
Exposure (ms)	1100
Rotation Step (deg)	0.24
Frame Averaging	ON (8)
Random Movement	ON (10)
Use 360 Rotation	YES
Geometrical Correction	ON

**Table 4:** Selected major reconstruction parameters.

Parameter	Value
Post-alignment	−1.00
Pixel Size (µm)	9.87947
Object Larger than FOV	OFF
Ring Artefact Correction	18
Beam Hardening Correction (%)	10
Threshold for defect pixel mask (%)	50
CS Static Rotation (deg)	5.00
Minimum for CS to Image Conversion	0.000
Maximum for CS to Image Conversion	0.040

## References

[1] Marquardt E. (1930). Geschleuderte Beton-und Eisenbetonrohre. Die Bautechnik.

[2] Achverdov I.N. (1961). Novoe v technologii železobetonnych centrifugirovannych rastrubnych trub. Beton i železobeton.

[3] Dilger W.H., Ghali A., Krishna Mohan Rao S.V. (1996). Improving the Durability and Performance of Spun-Cast Concrete Poles. PCI J..

[4] Dilger W.H., Krishna Mohan Rao S.V. (1997). High Performance Concrete Mixtures for Spun-Cast Concrete Poles. PCI J..

[5] Adesiyun A., Kamiński M., Kubiak J., Łodo A. Laboratory test on the properties of spun concrete. Proceedings of the Third Interuniversity Research Conference.

[6] Adesiyun A., Kamiński M., Kubiak J., Łodo A. (1996). Investigation of Spun-Cast Concrete Structure, School of Young Research Methodology of Concrete Structures.

[7] Wong R.C.K., Chau K.T. (2005). Estimation of air void and aggregate spatial distributions in concrete under uniaxial compression using computer tomography scanning. Cem. Concr. Res..

[8] Skarżyński Ł., Tejchman J. (2016). Experimental investigations of fracture process in concrete by means of X-ray micro-computed tomography. Strain.

[9] Du Plessis A., Olawuyi B.J., Boshoff W.P., Le Roux S.G. (2016). Simple and fast porosity analysis of concrete using X-ray computed tomography. Mater. Struct..

[10] Ostrowski K., Sadowski Ł., Stefaniuk D., Wałach D., Gawenda T., Oleksik K., Usydus I. (2018). The Effect of the Morphology of Coarse Aggregate on the Properties of Self-Compacting High-Performance Fibre-Reinforced Concrete. Materials.

[11] Schabowicz K., Jóźwiak-Niedźwiedzka D., Ranachowski Z., Kudela S., Dvorak T. (2018). Microstructural characterization of cellulose fibres in reinforced cement boards. Arch. Civ. Mech. Eng..

[12] Ponikiewski T., Gołaszewski J., Rudzki M., Bugdol M. (2015). Determination of steel fibres distribution in self-compacting concrete beams using X-ray computed tomography. Arch. Civ. Mech. Eng..

[13] Ponikiewski T., Katzer J., Bugdol M., Rudzki M. (2015). Steel fibre spacing in self-compacting concrete precast walls by X-ray computed tomography. Mater. Struct..

[14] Garboczi E.J. (2002). Three-dimensional mathematical analysis of particle shape using X-ray tomography and spherical harmonics: Application to aggregates used in concrete. Cem. Concr. Res..

[15] Kubiak J., Łodo A., Michałek J. (2015). Produkcja wirowanych żerdzi elektroenergetycznych w formach nieotwieranych podłużnie. Mater. Bud..

[16] Łydżba D., Rajczakowska M., Stefaniuk D., Kmita A. (2014). Identification of the carbonation zone in concrete using X-ray microtomography. Stud. Geotech. Mech..

[17] Torquato S. (2013). Random Heterogeneous Materials: Microstructure and Macroscopic Properties.

[18] Łydżba D. (2002). Zastosowania metody asymptotycznej homogenizacji w mechanice gruntów i skał. Prace Naukowe Instytutu Geotechniki i Hydrotechniki Politechniki Wrocławskiej. Monografie.

[19] Różański A. Sur la représentativité, la taille minimale du VER et les propriétés effectives de transport des matériaux composites aléatoires. Ph.D. Thesis.

[20] Hildebrand T., Rüegsegger P. (1997). A new method for the model-independent assessment of thickness in three-dimensional images. J. Microsc..

[21] Cała M., Cyran K., Kawa M., Kolano M., Łydżba D., Pachnicz M., Rajczakowska M., Różański A., Sobótka M., Stefaniuk D. (2017). Identification of Microstructural Properties of Shale by combined Use of X-ray Micro-CT and Nanoindentation Tests. Proced. Eng..

[22] Oliver W.C., Pharr G.M. (1992). An Improved Technique for Determining Hardness and Elastic Modulus Using Load and Displacement Sensing Indentation Experiments. J. Mater. Res..

[23] Miller M., Bobko C., Vandamme M., Ulm F.-J. (2008). Surface Roughness Criteria for Cement Paste Nanoindentation. Cem. Concr. Res..

[24] Struers-Ensuring Certainity. https://www.struers.com/en/Knowledge/Grinding-and-polishing#.

[25] (2015). ISO 14577-1:2015, Metallic Materials-Instrumented Indentation Test for Hardness and Materials Parameters-Part 1: Test Method ISO Central Secretariat.

[26] Doerner M.F., Nix W.D. (1986). A Method for Interpreting the Data from Depth-Sensing Indentation Instruments. J. Mater. Res..

[27] Fischer-Cripps A.C. (2007). Illustrative Analysis of Load-Displacement Curves in Nanoindentation. J. Mater. Res..

[28] Fischer-Cripps A., Nicholson D. (2004). Nanoindentation. Mechanical Engineering Series. Appl. Mech. Rev..

[29] Oliver W.C., Pharr G.M. (2004). Measurement of Hardness and Elastic Modulus by Instrumented Indentation: Advances in Understanding and Refinements to Methodology. J. Mater. Res..

[30] Sneddon I.N. (1948). Boussinesq’s problem for a rigid cone. Math. Proc. Camb. Philos. Soc..

[31] Constantinides G., Ravi Chandran K.S., Ulm F.-J., Van Vliet K.J. (2006). Grid Indentation Analysis of Composite Microstructure and Mechanics: Principles and Validation. Mater. Sci. Eng. A.

[32] Bobko C., Ulm F.-J. (2008). The Nano-Mechanical Morphology of Shale. Mech. Mater..

[33] Kanit T., Forest S., Galliet I., Mounoury V., Jeulin D. (2003). Determination of the Size of the Representative Volume Element for Random Composites: Statistical and Numerical Approach. Int. J. Solids Struct..

[34] Łydżba D., Różański A. (2014). Microstructure Measures and the Minimum Size of a Representative Volume Element: 2D Numerical Study. Acta Geophys..

[35] Berkovich E.S. (1951). Three Faceted Diamond Pyramid for Micro-Hardness Testing. Ind. Diam. Rev..

[36] Sorelli L., Constantinides G., Ulm F.-J., Toutlemonde F. (2008). The Nano-Mechanical Signature of Ultra High Performance Concrete by Statistical Nanoindentation Techniques. Cem. Concr. Res..

[37] Rajczakowska M., Stefaniuk D., Łydżba D. (2015). Microstructure Characterization by Means of X-Ray Micro-CT and Nanoindentation Measurements. Stud. Geotech. Mech..

[38] Al-Raoush R., Papadopoulos A. (2010). Representative Elementary Volume Analysis of Porous Media Using X-Ray Computed Tomography. Powder Technol..

[39] Cnudde V., Cwirzen A., Masschaele B., Jacobs P.J.S. (2009). Porosity and Microstructure Characterization of Building Stones and Concretes. Eng. Geol..

[40] Peyton R.L., Haeffner B.A., Anderson S.H., Gantzer C.J. (1992). Applying X-ray CT to Measure Macropore Diameters in Undisturbed Soil Cores. Geoderma.

[41] Feldkamp L.A., Davis L.C., Kress J.W. (1984). Practical Cone-Beam Algorithm. J. Opt. Soc. Am. A JOSAA.

[42] Rodet T., Noo F., Defrise M. (2004). The Cone-Beam Algorithm of Feldkamp, Davis, and Kress Preserves Oblique Line Integrals. Med. Phys..

